# New Perspective for Drug–Drug Interaction in Perioperative Period

**DOI:** 10.3390/jcm12144810

**Published:** 2023-07-21

**Authors:** Abigail Silva, Bárbara Costa, Irene Castro, Joana Mourão, Nuno Vale

**Affiliations:** 1 OncoPharma Research Group, Center for Health Technology and Services Research (CINTESIS), Rua Doutor Plácido da Costa, 4200-450 Porto, Portugal; abigailsilva@outlook.pt (A.S.); b.c.211297@gmail.com (B.C.); irene.castro@ipoporto.min-saude.pt (I.C.); 2 CINTESIS@RISE, Faculty of Medicine, University of Porto, Alameda Professor Hernâni Monteiro, 4200-319 Porto, Portugal; joanamourao@med.up.pt; 3 Department of Anesthesiology and Intensive Care Medicine, Instituto Português de Oncologia do Porto (IPO-Porto), 4200-072 Porto, Portugal; 4 Department of Anesthesiology, Centro Hospitalar Universitário de São João, Alameda Professor Hernâni Monteiro, 4200-319 Porto, Portugal; 5 Surgery and Physiology Department, Faculty of Medicine, University of Porto, Rua Doutor Plácido da Costa, 4200-450 Porto, Portugal; 6 Department of Community Medicine, Health Information and Decision (MEDCIDS), Faculty of Medicine, University of Porto, Rua Doutor Plácido da Costa, 4200-450 Porto, Portugal

**Keywords:** drug–drug interactions, perioperative period, pharmacokinetic interactions, pharmacodynamic interactions, patient safety

## Abstract

In this review, we aim to discuss current information on drug interactions in the perioperative period. During this period, patients receive several drugs that may interact with each other and affect the efficacy and safety of the treatment. There are three types of drug interactions: pharmacodynamic, pharmacokinetic, and pharmaceutical. It is important to recognize that drug interactions may increase the toxicity of the drug or reduce its efficacy, increasing the risk of complications in the perioperative period. This review describes the most commonly used perioperative drugs approved by the FDA and some of the described interactions between them. Thoroughly reviewing a patient’s medication list and identifying potential interactions are essential steps in minimizing risks. Additionally, vigilant monitoring of patients during and after surgery plays a pivotal role in early detection of any signs of drug interactions. This article emphasizes the significance of addressing DDIs in the perioperative period to ensure patient well-being and advocates for the implementation of careful monitoring protocols to promptly identify and manage potential interactions.

## 1. Introduction

**Figure 1:**
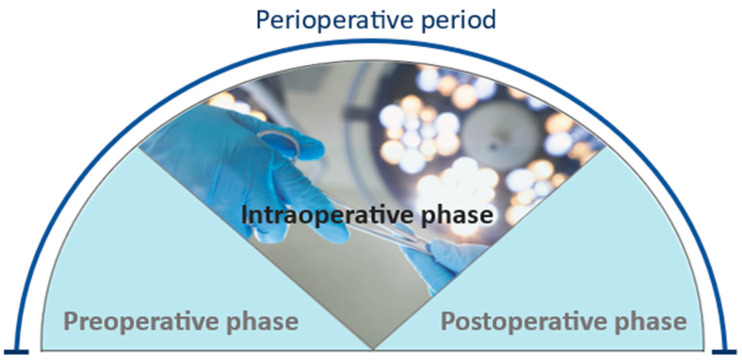
Representation of the perioperative period in its three constituent phases, made in Adobe Illustrator on 25 May 2023.

The perioperative period is the whole period including the preoperative period, the intraoperative phase, and the postoperative phase (Figure 1). This process provides the patient with all integrated care, from emotional and physical preparation and preliminary studies for a good operation, the contemplation of surgery, and the monitoring of recovery to prevent complications. The aim of perioperative care is to provide a healthier environment for patients before, during, and after the operation [1,2].

This perioperative management includes the use of drugs and opioids for better pain management. The use of all these drugs and opioids can lead to drug–drug interaction (DDIs), which is the effect produced by a combination of two or more drugs, and these DDIs can have a negative effect when one drug alters the effect of the other or when a disease alters the pharmacokinetics or pharmacodynamics of a drug leading to an adverse drug reaction (ADR), a harmful reaction to a medicine given at the correct dose [3,4,5,6,7]. ADRs represent an important clinical issue, as they are a worthy cause of morbidity and mortality and increase health costs, but as medical knowledge advances, various methods have been developed to assess potentially harmful DDIs, using electronic databases that report the prevalence of drug interactions as a tool [4,8].

These drug–drug interactions also happen in the operation, during anesthesia, which is administered in the intraoperative phase. Anesthetists are often faced with anesthetic drug interactions and make therapeutic decisions involving these same drugs because the desired effects of anesthesia are obtained from a single drug or from the combination of several drugs, so the anesthetist must be aware of the dose adjustment and learn how to manage their perioperative course.

When drugs are combined for anesthesia, all must contribute to the same effect, and when a combination is used, the dose of each individual anesthetic drug is reduced compared to when the doses of individual drugs are used to achieve the same effect. These interactions between drugs may decrease the severity or incidence of effects without impairing the desired effects; however, despite this, excessive administration of anesthetics may hinder recovery or increase the risk of adverse effects, and these drug interactions can be distinguished by three ways, additivity, supra-additivity (or synergism), and infra-additivity (or antagonism) [9,10].

**Figure 2:**
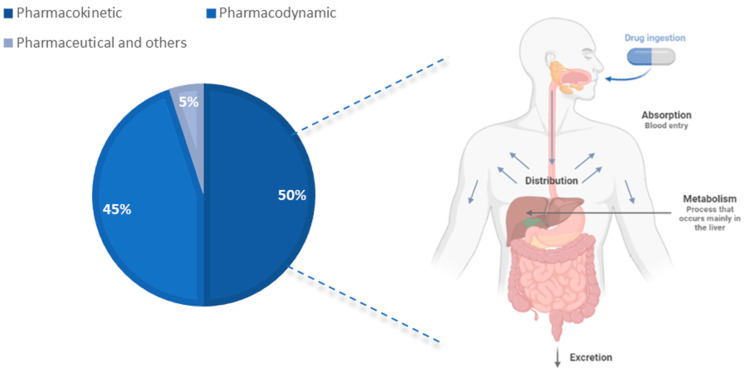
Graph illustrating the different interactions that can occur in the body between medicines. Five percent of interactions are pharmaceutical and unknown; forty five percent are pharmacodynamics; and fifty percent are pharmacokinetic. These pharmacokinetic interactions are divided into four phases, absorption, distribution, metabolism, and excretion, respectively, in the order they occur in the body, made in BioRender on 20 May 2023.

Indeed, anesthetic techniques depend on beneficial drug–drug interactions, as a good combination of these leads to increased efficacy and safety of drug treatment. These drugs can interact in three separate ways, pharmaceutical, pharmacodynamic, or pharmacokinetic [4]. Therefore, the success of the DDIs and the anesthesia depends on these characteristics (Figure 2).

Pharmacodynamic interactions are the most predictable and therefore the most easily avoided. These occur when two or more drugs interact at the same site or receptor in the body, and this type of drug interaction can increase or decrease the effectiveness, increase the toxicity, or reduce its effectiveness. Common examples of pharmacodynamic interactions include the use of opioids and benzodiazepines, which can cause respiratory depression. The other two interactions, in contrast, are more difficult to predict, even with all the knowledge of pharmacokinetics and drug properties already known.

On the other hand, pharmaceutical drug interactions usually occur before the drug is administered and occur by chemical or physical reactions. In chemical reactions, there may be salt formation, oxidation-reduction, acid-base, hydrolysis, or epimerization, and in physical reactions there may be adsorption/absorption and emulsion breaking.

In pharmacokinetic interactions, one drug alters the absorption, distribution, or elimination of another drug. This type of interaction can affect the efficacy or toxicity of the medicine, as well as the duration of the medicine’s effect, and these interactions occur due to the inhibition or induction of cytochrome P450 enzyme [11].

**Figure 3:**
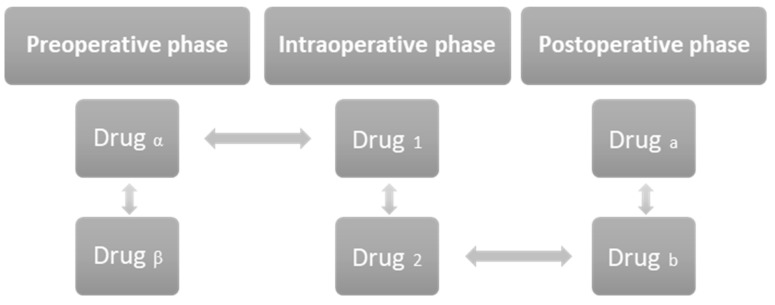
Figure representing the types of interactions that occur between drugs and these interactions may be vertical, when they occur between drugs administered to the patient in the same phase of the perioperative period, or vertical interactions, which occur between drugs that are administered to the patient in different phases of the perioperative period. The drugs name described in the figure are illustrative.

The different drugs given during the three phases of the perioperative period interact in different ways with each other. They may interact with each other in each phase or interact with the drugs given in the other two phases (Figure 3).

Different drugs were administered during the three phases of the perioperative phase (Table 1) [12,13,14,15,16,17,18,19,20,21,22,23,24,25,26,27]. We investigated about their clinical function, adverse reactions, and possible interactions with other drugs as described in the following parts of this review. 

## 2. Vertical Interactions

### 2.1. Preoperative Period

Drugs used in the pre-operative phase can have various effects on the body. Those effects can be increased or decreased according to the interaction with other drugs, and a new effect can sometimes occur. There are harmful drug interactions in the pre-operative phase.

The most common interactions result from non-steroidal anti-inflammatory drugs (NSAIDs) and anticoagulants, which may increase the risk of bleeding during and after surgery [28], benzodiazepines and opioids, which can increase sedative/hypnotic and respiratory depressant effects, sometimes leading to respiratory complications during surgery, and between antihypertensives and anesthetics. The use of antihypertensives before surgery may increase the risk of low blood pressure (hypotension and cerebral/myocardial/kidney hypoperfusion) during surgery, leading to an increased postoperative morbidity and adverse events.

#### 2.1.1. Methadone vs. Benztropine

Methadone is a synthetic opioid medication used for the long-term management and treatment of opioid addiction withdrawal symptoms, as well as for chronic pain relief. In the treatment process, other medications may be combined with methadone due to the common occurrence of comorbidities among drug addicts. These additional medications can include psychotropic drugs, antibiotics, anticonvulsants, and antiretroviral drugs. Methadone has a prolonged elimination half-life of 24 to 36 h, and when methadone is coadministered with weak to strong CYP3A inhibitors or a moderate CYP3A4 inducer, its exposure in the body may either decrease or remain unchanged. However, methadone exposure is reduced when combined with CYP2B6 inducers [29,30].

Benzodiazepines are a class of drugs that enhance neurotransmission at GABAergic synapses. They are commonly used as anti-anxiety medications and as adjunctive treatment for various neurological and psychiatric disorders [31,32]. However, their misuse is prevalent among individuals taking methadone. Benzodiazepines can amplify the euphoric effects of opioids, alleviate withdrawal symptoms, moderate cocaine highs, potentiate alcohol effects, and modulate withdrawal states. They typically induce a mild to moderate depression of the central nervous system, with deep coma requiring assisted ventilation being rare and suggesting the presence of other toxic substances. The severity of CNS depression depends on factors such as the dosage, the patient’s age, and clinical condition prior to ingestion and the co-ingestion of other central nervous system depressants [32]. In severe cases of overdose, benzodiazepines can occasionally cause cardiovascular and pulmonary toxicity. While not generally life-threatening, benzodiazepine intoxication can pose a life-threatening risk in certain situations or populations with underlying health conditions [33,34].

The combination of methadone and benzodiazepines can lead to pharmacokinetic interactions. Drugs with depressant effects, including benzodiazepines, opioids, alcohol, and antipsychotics, should be closely supervised, and monitored. The interaction between benzodiazepines and methadone can result in severe side effects, such as excessive sedation, respiratory depression, and coma, particularly when both drugs are used together. Since both medications have the potential for physical and psychological dependence, this interaction is of particular concern [29,35].

#### 2.1.2. Tramadol vs. Benzodiazepine

Tramadol is the most commonly prescribed opioid analgesic medication. It acts as a synthetic partial agonist on μ-opioid receptors and inhibits the reuptake of norepinephrine and serotonin. However, the way tramadol is metabolized, and the resulting metabolites depend on interactions with cytochrome P450 enzymes. The half-life of tramadol is approximately 6–8 h, which means that after 6–8 h of taking the drug, the concentration of tramadol in the bloodstream is reduced by half [36,37,38].

It is available in various formulations, such as drops, capsules, sustained-release tablets for oral use, suppositories for rectal use, and solutions for intramuscular, intravenous, and subcutaneous injection. When taken orally, tramadol is rapidly and almost completely absorbed. Sustained-release tablets gradually release the active ingredient over a period of 12 h, reaching peak concentrations after approximately 4.9 h. They have a bioavailability of 87–95% compared to capsules [36]. Tramadol is quickly distributed throughout the body, with about 20% of it binding to plasma proteins. The main metabolic pathways of tramadol involve O- and N-demethylation, as well as conjugation reactions forming glucuronides and sulfates. Tramadol and its metabolites are primarily eliminated through the kidneys.

Due to its inhibitory effects on serotonin and norepinephrine reuptake, tramadol has unique adverse effects. These can include the potential for serotonin syndrome, seizures, dizziness, nausea, constipation, and headache, and, if left untreated, they can lead to serious health consequences and even mortality [39,40]. Additionally, tramadol can interact with other medications, particularly antidepressants and benzodiazepines. The interaction with benzodiazepines is noteworthy because both drugs act as central nervous system depressants, and when taken together, they can enhance each other’s sedative and depressant effects.

Studies have shown that premature deaths in patients who abuse opioids are often associated with the ingestion of other central nervous system depressants, such as benzodiazepines. Cases have been reported involving various medications, including tramadol, when taken concomitantly with different benzodiazepines. The exact reasons for these interactions are not fully understood, but they may be attributed to pharmacodynamics and pharmacokinetics [41].

#### 2.1.3. Paracetamol vs. Tramadol

Paracetamol (acetaminophen) is the most used medication worldwide, widely prescribed for both acute and chronic pain relief. However, there are certain areas where the evidence of potential harm is stronger, particularly when paracetamol is used chronically. In such cases, it is advisable for clinicians to discuss these side effects with patients beforehand. The evidence particularly points to an increased risk of gastrointestinal bleeding and a slight (~4 mmHg) elevation in systolic blood pressure, both of which show a degree of dose dependence. Paracetamol is often involved in intentional or unintentional overdoses, leading to severe liver injury and acute liver failure (ALF) [42,43].

After ingestion, paracetamol is rapidly absorbed, and therapeutic levels are reached within 40 to 120 min. Peak levels are achieved within 2 to 4 h. The elimination half-life of paracetamol is 2 to 4 h. The potentially toxic dose for adults is only about 10 times higher than the usual therapeutic dose of 650 to 1000 mg. As a result, paracetamol is one of the most common drugs involved in accidental or intentional overdoses. Its toxicity is dose-related, with most cases of acute liver failure occurring with daily doses exceeding 10 g (>150 mg/kg). Moderate chronic use of approximately 4 g/day may lead to mild and transient liver enzyme elevations in healthy individuals, but in rare cases, it can result in acute liver failure. Chronic alcohol use or the use of certain drugs like barbiturates or isoniazid can increase susceptibility to paracetamol toxicity [43].

In the liver, 52% to 57% of ingested paracetamol is converted to glucuronide conjugates, while 30% to 44% is converted to sulfate conjugates. These conjugates are non-toxic, water-soluble, and rapidly excreted in the urine. Approximately 5% to 10% of ingested paracetamol undergoes metabolism via the cytochrome P-450 system, primarily involving the isoenzyme P450 2E1, with contributions from 1A2, 3A4, and 2A6. P450 2E1 is the same isoenzyme responsible for metabolizing ethanol and can be induced by regular alcohol consumption, leading to increased metabolism of paracetamol through this pathway [43,44]. Metabolism of paracetamol via the cytochrome P450 pathway produces N-acetyl-p-benzoquinone imine (NAPQI), which depletes the body’s stores of glutathione. When glutathione becomes depleted, NAPQI binds to intracellular proteins containing sulfhydryl groups, causing cellular dysfunction and liver necrosis.

Tramadol is a commonly prescribed opioid medication for various types of pain. Unlike traditional opioids, it acts not only as a µ-opioid agonist but also through other mechanisms, including the reuptake inhibition of serotonin and norepinephrine. Although tramadol is less potent than morphine and similar opioids, it is used to alleviate moderate to severe pain [37].

Acetaminophen and tramadol are frequently prescribed together to manage pain and are sometimes combined in a single pill or capsule. The combination of these medications can be more effective in relieving pain compared to using each medication alone. However, it is important to exercise caution when taking them together as there can be interactions between these drugs that increase the risk of side effects. These side effects may include drowsiness, headache, and respiratory depression associated with tramadol. Added to these effects, paracetamol is a distress and fever reducer that, when taken in high doses, can cause liver damage. That is, when taken together, tramadol and acetaminophen may increase the risk of liver damage, especially if the recommended doses are exceeded [45].

### 2.2. Postoperative Period

Just like drugs used in the pre-operative phase, those used in the postoperative phase can have various effects on the body, and those effects can be increased or decreased according to the interaction with other drugs, and a new effect can even occur. There are harmful drug interactions in the postoperative phase too. The most common drugs used in this phase are (1) painkillers, to relieve the pain, such as opioids, paracetamol, and ibuprofen; (2) anti-inflammatory drugs, used to reduce inflammation and swelling; (3) antibiotics, to prevent infections; (4) anticoagulants, to prevent blood clots forming; and (5) antiemetics, to treat and prevent nausea and vomiting caused after the surgery [46]. It is essential to recognize that interactions among these drugs may occur during the postoperative phase, potentially leading to harmful drug interactions. Healthcare professionals must exercise vigilance when selecting and administering these medications to ensure optimal patient outcomes. Close monitoring of patients and prompt identification of any signs of drug interactions are necessary to minimize risks and promote a successful recovery.

#### 2.2.1. Celecoxib vs. Morphine

Celecoxib, a selective COX-2 inhibitor, has been used for over two decades as a medication with anti-inflammatory, analgesic, and antipyretic properties. It belongs to the class of nonsteroidal anti-inflammatory drugs (NSAIDs) and is primarily prescribed for the treatment of pain and inflammation. On the other hand, morphine is an opioid analgesic used to alleviate moderate to severe pain. When celecoxib and morphine are used together, there is an increased risk of side effects such as gastrointestinal bleeding, kidney problems, high blood pressure, and heart failure. Therefore, unless prescribed by a healthcare professional and closely supervised, the combination of these medications should be avoided [47].

Celecoxib is moderately absorbed when taken orally, with peak plasma drug concentration occurring after 2 to 4 h. The extent of absorption is not well known. It binds extensively to plasma albumin and has a volume of distribution of approximately 455 ± 166 L in humans. Celecoxib is eliminated through biotransformation into carboxylic acid and glucuronide metabolites, which are excreted in urine and feces. Only a small percentage of the drug (2%) is excreted unchanged in the urine. The primary metabolism of celecoxib occurs through the cytochrome P450 (CYP) 2C9 isoenzyme, and it has an elimination half-life of approximately 11 h in healthy individuals. Racial differences in drug disposition and changes in pharmacokinetics have been observed in the elderly population. In patients with chronic renal insufficiency (glomerular filtration rate 2.1 to 3.6 L/h or 35 to 60 mL/min), plasma concentrations (AUC) of celecoxib appear to be 43% lower compared to individuals with normal renal function, with a 47% increase in apparent clearance. Since celecoxib is metabolized by CYP2C9, caution is needed when coadministering it with other drugs that are substrates or inhibitors of this enzyme [48].

Morphine, the most commonly used μ-opioid analgesic for acute and chronic pain, is seen as the standard against which new analgesics are compared. Understanding the pharmacokinetics of morphine is crucial for its safe and effective use in patients with varying degrees of organ disfunction. Peak plasma levels are reached within 15–20 min after intramuscular or subcutaneous administration and within 30–90 min after oral administration. However, peak levels after oral administration are much lower than those achieved through parenteral routes, as oral morphine undergoes extensive first-pass metabolism in the liver [49,50].

If a patient needs to take both celecoxib and morphine, the healthcare provider may adjust the dosage of each drug to minimize the risk of drug interaction. It is important to strictly adhere to the prescribed dosages for each medication to avoid potentially harmful drug interactions [50].

#### 2.2.2. Corticosteroids vs. Ibuprofen

Ibuprofen belongs to the class of chiral nonsteroidal anti-inflammatory drugs (NSAIDs) known as 2 arylpropionic acids (2-APA). When taken orally, ibuprofen is rapidly and completely absorbed. The extent of ibuprofen’s absorption, as measured by the area under the plasma concentration–time curve (AUC), is dependent on the dosage. Common side effects of oral ibuprofen, which occur in more than 1 in 100 individuals, include headaches, dizziness, nausea, vomiting, flatulence, and indigestion. Ibuprofen is rapidly absorbed, and peak serum levels are reached within one to two hours after administration. It has a relatively short half-life of 1.8 to 2 hours. Ibuprofen primarily binds to plasma albumin and has a volume of distribution of 0.12 L/kg in adults. It also accumulates in synovial fluid, which is the proposed site of action for NSAIDs. When ibuprofen is taken at doses greater than 600 mg, the unbound fraction of the drug increases, leading to enhanced clearance. Metabolism of ibuprofen occurs through biotransformation into glucuronide conjugate metabolites, which are eliminated in urine and feces. Approximately 45–79% of the drug is excreted as metabolites, while less than 1% remains unchanged. The maximum recommended daily dose of ibuprofen is 3200 mg, and its therapeutic range is broad, ranging from 10 to 50 mg/L, with high concentrations (>100 mg/L) considered toxic [51,52].

When corticosteroids are used concomitantly with ibuprofen, there is an increased risk of gastrointestinal side effects such as ulcers, bleeding, or perforations in the stomach or intestines. Corticosteroids can cause gastric irritation, while ibuprofen can reduce gastric mucus production and compromise the integrity of the gastric mucosa, rendering it more susceptible to stomach acid. It is essential to consult with your doctor or pharmacist before using these medications together, particularly if you have a history of stomach or intestinal ulcers, bleeding, or kidney or liver problems. If these two drugs are prescribed simultaneously, the dosage should be adjusted, and protective measures for the stomach should be recommended, such as taking the medications with food or using stomach-protective medications [53].

### 2.3. Intraoperative Period

Anesthesia is a treatment used during surgical and/or diagnostic procedures, and its main purpose is deep hypnosis, and amnesia is to not let the patient feel pain or sensation during the procedure and to prevent the physiological repercussions of aggression. The drugs used in this process are called anesthetics. There are distinct types of anesthesia. It can be local, regional (nervous plexus, epidural, or spinal), or general. Before surgery, everything about the patient medical historic records is taken into consideration, such as comorbidities, adverse reactions to previous anesthetics, current prescriptions, allergies, use of alcohol, smoking, or abuse of drugs.

Combined anesthesia is a technique where the general and regional anesthesia are combined and used together to minimize the side effects and maximize both efficacy and safety. To have a more effective and safer anesthesia, there are different types of combinations of combined anesthesia that can be used and made, taking in account their uniqueness in view of the patient and the procedure, such as intravenous general anesthesia with regional anesthesia, intravenous general anesthetic with inhalational anesthesia, intravenous general anesthetic with neuromuscular blockade, and regional anesthesia with conscious sedation [54,55].

#### 2.3.1. Propofol vs. Isoflurane

Isoflurane is an FDA-approved volatile anesthetic used for inducing and maintaining general anesthesia. It works by inhibiting specific receptors in the central nervous system, including GABA, glycine, and NMDA receptors, which helps to achieve the desired sedation and amnesia during surgery. Careful titration is necessary to manage the patient’s hemodynamics because isoflurane can cause significant drops in blood pressure through peripheral vasodilation, particularly in hypovolemic patients [56]. It is important to note that isoflurane, like other halogenated volatile anesthetics, can trigger malignant hyperthermia in susceptible individuals, especially those with a personal or family history of the condition.

Isoflurane has several effects on the cardiovascular system. It reduces the cardiac output by approximately 20% below the control value 15 min after anesthesia. This is primarily due to the dilation of peripheral blood vessels, leading to dose-dependent hypotension. In older patients, isoflurane can have a slight negative impact on heart muscle contractility, which may result in cardiac insufficiency or myocardial hypoxia, particularly in patients with pre-existing cardiac damage. Additionally, exposure to high concentrations of isoflurane can decrease the contractility of the uterus muscle. It is worth noting that respiration is not stimulated by hypoxia during isoflurane anesthesia [57,58].

Propofol is an intravenous hypnotic drug used for inducing and maintaining sedation and general anesthesia. It achieves its effects by enhancing the inhibitory neurotransmitter γ-aminobutyric acid (GABA) at the GABAA receptor. Propofol is widely used due to its favorable drug profile, but it can cause disturbances in cardiopulmonary physiology. Serious allergic reactions, including anaphylaxis, are possible, and immediate medical attention is necessary if symptoms such as rash, itching, hoarseness, difficulty breathing, or swelling occur. Propofol infusion syndrome is another potential complication, which can lead to more severe problems such as high potassium levels, elevated fat or cholesterol in the blood, rhabdomyolysis, enlarged liver, kidney failure, or heart failure. This often happens in prolonged infusions, which is not usual in the intraoperative period, essentially in intensive care units. Dizziness, drowsiness, blurred vision, confusion, and other side effects may also occur [59,60,61].

Propofol is an intravenous anesthetic used for induction and maintenance of anesthesia during surgery. When propofol and isoflurane are used together, the effects of both drugs are enhanced, resulting in a stronger anesthetic and sedative effect. However, both propofol and isoflurane depress the central nervous system, and when combined with other drugs that have similar effects (such as opioids and other inhaled anesthetics), there can be an increased risk of problems associated with central nervous system depression [59].

It is important to be aware that combining propofol and isoflurane can increase the risk and severity of central nervous system depression. Medical professionals should carefully consider this when administering these drugs together [62].

#### 2.3.2. Propofol vs. Remifentanil

The combination of these two drugs, remifentanil and propofol, is usual. Remifentanil is, just like propofol, a continuous intravenous drug, which provides analgesia and helps to facilitate brief and painful procedures. The stability of these two drugs combined depends on their proportion, time, and the receptor, and if these variables are evaluated, remifentanil and propofol can be used together. Remifentanil can also be used in the postoperative period [63].

Studies demonstrated that propofol pharmacokinetics remains unaffected when this drug is mixed with remifentanil. Studies also show that propofol reduces the central volume of distribution (41%), clearance (41%), and removal (15%) of remifentanil. Furthermore, to obtain a balanced dose of remifentanil and propofol, it is necessary to change the volume of distribution of remifentanil, which can lead to adverse situations such as bradycardia and hypotension during the surgery [29,64,65,66].

#### 2.3.3. Propofol vs. Sevoflurane

Sevoflurane is a commonly used inhalation anesthesia, particularly in pediatric surgery. Prolonged exposure to this drug has been found to be neurotoxic to the brain in animal studies. Although human studies are not as conclusive, research has shown that children under the age of three who underwent surgery were 60% more likely to be diagnosed with developmental and behavioral disorders, with continuous anesthesia exposure being one of the associated risk factors. Studies in mice and monkeys have demonstrated the adverse effects of sevoflurane, such as decreased social interaction in rats exposed to maternal anesthesia, increased anxiety in adolescent monkeys exposed to sevoflurane during infancy, and increased risk of cognitive dysfunction and attention-deficit/hyperactivity disorder in adult mice exposed to sevoflurane during the neonatal phase [67,68,69,70].

Sevoflurane induces a dose-dependent reduction in blood pressure and cardiac output primarily by reducing systemic vascular resistance. It can also cause airway irritation, leading to coughing, apnea, and laryngospasm. Other effects include bronchodilation, blunting of the hypoxia/hypercapnia ventilatory response, increased cerebral blood flow and intracranial pressure, and reduced cerebral metabolic rate. Common side effects of sevoflurane include hypotension, emergence delirium, agitation, nausea, vomiting, tachycardia, bradycardia, hypertension, laryngospasm, and apnea. Rare but serious adverse reactions may include anaphylaxis, cardiac arrhythmias, increased intracranial pressure, hepatotoxicity, electrolyte disturbances, and malignant hyperthermia [28,68,71,72,73].

When sevoflurane is used in combination with propofol, lower concentrations of sevoflurane may be required. The combined use of these drugs prolongs the recovery time for the patient due to the deeper anesthesia, but it also lowers certain risks. For example, while sevoflurane alone can increase blood pressure when combined with propofol, it can counteract this effect and lower blood pressure. Hemodynamically, the combined use (occurs in few situations) of sevoflurane and propofol is advantageous, but it can be disadvantageous in terms of the occurrence of convulsions and recovery time [74,75]. It is important to note that combining propofol with sevoflurane can increase the risk or severity of central nervous system depression [74].

#### 2.3.4. Propofol vs. Fentanyl

Fentanyl is an opioid drug that is 100 to 300 times stronger than morphine. It is primarily used for pain management and can also be used in combination with other drugs for anesthesia. Fentanyl can also be administered intravenously, through transdermal patches, or orally. It provides cardiovascular stability in patients and is commonly used as an adjunct to anesthesia. The hepatic metabolism of fentanyl occurs through the CYP450 enzyme system, specifically CYP3A4, and it has a half-life of 3 to 7 h. The drug is excreted through urine (75%) and feces (9%) [76,77].

The side effects of fentanyl are similar to those of heroin, including euphoria, confusion, respiratory depression, drowsiness, nausea, visual disturbances, dyskinesia, hallucinations, delirium (including narcotic delirium), analgesia, constipation, narcotic ileus, muscle rigidity, addiction, loss of consciousness, hypotension, coma, and even death. Concurrent use of alcohol and other drugs such as cocaine or heroin can intensify the side effects of fentanyl, leading to complex clinical situations that can be challenging to manage. These substances, when combined, create undesirable conditions that complicate the patient’s prognosis [76].

Fentanyl is relatively contraindicated in the following situations: patients undergoing biliary tract surgery, as it may impede hepatic drug elimination; patients with respiratory depression or obstructive airway diseases such as asthma, COPD, obstructive sleep apnea, and obesity hypoventilation syndrome (Pickwickian syndrome) and liver failure; patients with known intolerance to fentanyl or other morphine-like drugs, including codeine or any components in the formulation; and patients with known hypersensitivity or anaphylaxis to fentanyl or common drug delivery excipients such as sodium chloride and sodium hydroxide [76].

Despite the analgesic efficacy and balanced anesthesia provided by propofol and fentanyl, the combination of these drugs has been associated with bradycardia and asystole in patients with repeated use. Co-administration of propofol and fentanyl has also been shown to increase intraoperative blood loss to a greater extent compared to the combination of propofol. This significant blood loss during surgery is a serious concern as it may necessitate blood transfusion, which in turn carries an increased risk of postoperative bacterial infections. Also, fentanyl may increase the sedative activities of propofol [78,79].

#### 2.3.5. Ketamine vs. Vecuronium

Ketamine is a potent anesthetic that acts as an NMDA receptor antagonist. It interacts with various receptors, including NMDA receptors, opioid receptors, monoaminergic receptors, muscarinic receptors, and voltage-sensitive calcium ion channels. Unlike other general anesthetic agents, ketamine does not interact with GABA receptors. It is rapidly absorbed with a bioavailability of approximately 93%, but after first-pass metabolism, only 17% of the administered dose is absorbed. Ketamine distributes rapidly in the body, with a distribution half-life of 1.95 min. Preclinical studies have shown that ketamine, when used for longer than 3 h, can increase apoptosis in the developing brain, leading to cognitive deficits. However, toxicity studies regarding carcinogenesis have not been conducted, and ketamine has shown to be clastogenic without effects on fertility [80].

Ketamine can be used alone or in combination with other drugs. It is a sedative and dissociative anesthetic that acts on NMDA receptors. Adverse effects of ketamine may include disorientation, confusion, loss of motor coordination, dizziness, nausea, vomiting, and increased blood pressure, heart rate, breathing, or body temperature, as well as changes in sensory perceptions such as visual or auditory hallucinations and a sense of detachment from oneself, the surroundings, or the environment.

Vecuronium is a neuromuscular blocking agent used in general anesthesia to induce muscle relaxation during surgery. It works by blocking the transmission of nerve impulses. The use of vecuronium increases the risk of respiratory depression, especially when combined with ketamine, as it can enhance the effects of sympathetic nervous system stimulation [81]. Additionally, the use of vecuronium is associated with a significant prolongation of the duration of muscle relaxation. Adverse effects of vecuronium may include prolonged paralysis, bronchospasm/respiratory depression, apnea, anaphylaxis/hypersensitivity reactions, and other less common events such as hypotension, edema, sinus tachycardia, erythema, urticaria, flushing, pruritus, and skin rash [82].

It is important to note that combining ketamine with vecuronium can increase the risk or severity of central nervous system depression.

#### 2.3.6. Dexmedetomidine vs. Flurbiprofen

An alpha-2 agonist called dexmedetomidine is frequently used to sedate patients during medical operations. It reduces sympathetic activity and blocks the release of norepinephrine by attaching to alpha-2 adrenoceptors, which lowers blood pressure and heart rate and prevents the transmission of pain signals. This drug is essential for sedating non-intubated patients prior to or during procedures, as well as intubated and mechanically ventilated patients in intensive care settings. In order to improve analgesia during anesthesia, dexmedetomidine is frequently coupled with other anesthetic medications, such as parecoxib, methoxyphene, midazolam, dezocine, and NSAIDs [83]. No significant pharmacokinetic (PK) interactions between dexmedetomidine and propofol, midazolam, isoflurane, or fentanyl have been seen when taken at indicated target doses. However, a case report suggests that dexmedetomidine infusion may inhibit CYP3A4, causing tacrolimus levels to rise by four times [84]. Additionally, the concomitant use of antidepressants with dexmedetomidine may impact its pharmacokinetics (PK) and/or pharmacodynamics (PD), potentially intensifying its sedative effect (e.g., benzodiazepine).

Flurbiprofen, an NSAID, is used to relieve symptoms of arthritis, such as inflammation, swelling, stiffness, and joint pain. It exerts its anti-inflammatory effect by reversibly inhibiting cyclooxygenase (COX), an enzyme involved in prostaglandin synthesis. Flurbiprofen inhibits both COX-1 and COX-2, effectively decreasing the concentration of prostaglandins responsible for inflammation, pain, swelling, and fever. It is known for its potent prostaglandin inhibitory activity among NSAIDs [85].

In patients undergoing laparoscopic-assisted vaginal hysterectomy, a recent study examined the impact of combination dexmedetomidine and flurbiprofen axetil therapy on remifentanil-induced hyperalgesia (LAVH). Remifentanil was administered intra-operatively to Group H; Remifentanil plus a continuous infusion of dexmedetomidine was administered intra-operatively to Group HD; and Remifentanil, Flurbiprofen Axetil, and Dexmedetomidine were administered intra-operatively to Group HDF. The findings revealed that Group H had much lower mechanical pain thresholds than Groups HD and HDF. Additionally, compared to the other two groups, Group H had a larger area of secondary hyperalgesia at the incision site. Both the total amount of hyanil consumed and the visual analog scale (VAS) ratings were considerably greater in Group H. Dexmedetomidine and flurbiprofen axetil worked well to reduce the effects of remifentanil-induced hyperalgesia in mice [86].

Another study looked at the impact of dexmedetomidine and flurbiprofen axetil on immunological function and postoperative analgesia in lung cancer patients following radical surgery. The findings demonstrated that, in comparison to flurbiprofen axetil alone, the combination of dexmedetomidine and flurbiprofen axetil considerably reduced mean arterial pressure and heart rate. At 6 h and 12 h following the procedure, the VAS score and Bruggrmann comfort scale (BCS) score were lower in the combination group. Sufentanil dosage and analgesia pump pressing intervals were also significantly lower in the combo group. Higher amounts of NK cells, CD3+ T cells, and CD4+/CD8+ ratios were also produced by the combination, indicating enhanced immune function [87]. In summary, the combination of dexmedetomidine and flurbiprofen axetil shows promising results in preventing hyperalgesia and improving postoperative pain control and immune function in patients undergoing hysterectomy and lung cancer surgery, respectively.

## 3. Horizontal Interactions

### 3.1. Benzodiazepine vs. Ketamine

The use of benzodiazepines is frequent before the induction of anesthesia, as it helps to relieve anxiety and sedation. However, the concomitant use of benzodiazepines with ketamine may result in a decrease in the effectiveness of ketamine, as benzodiazepines can antagonize its psychedelic and analgesic effects. Despite the positive effect of antagonizing the psychedelic effect, it can antagonize the analgesic effect. Additionally, the use of these two drugs together may result in increased side effects such as respiratory depression and apnea. In elderly or debilitated patients, the situation can be problematic and result in sleep apnea, as these patients may have a less good response to medications. Basically, this interaction can vary according to the type and dose of benzodiazepines and ketamine used. It is a complex interaction and must be adapted to the type of patient [88,89,90].

### 3.2. Methadone vs. Ketamine

The concurrent administration of methadone and ketamine can result in a pharmacological interaction that increases the potential for side effects and toxicity. Ketamine has the ability to enhance the depressant effects of methadone, leading to a decrease in respiratory rate and blood pressure, particularly at higher doses [91]. Furthermore, the combination of methadone and ketamine may elevate the risk of psychological side effects, including hallucinations, confusion, and delirium. Due to these concerns, it is crucial to exercise caution when using methadone and ketamine together, closely monitoring for signs of adverse reactions. The concurrent use of ketamine and methadone may heighten side effects such as dizziness, drowsiness, confusion, difficulty concentrating, excessive sedation, and respiratory depression. Elderly individuals, in particular, may experience impairment in thinking, judgment, and motor coordination as a result [92,93,94].

### 3.3. Corticosteroids vs. Benzodiazepine

Corticosteroids are commonly prescribed for various medical conditions due to their broad range of effects on the body. While they have therapeutic benefits, corticosteroids are also known for their dose- and duration-dependent toxicities. Common adverse effects observed in patients treated with corticosteroids include hyperglycemia, susceptibility to superinfections, and prolonged hospital stays [95,96].

Dexamethasone and betamethasone are long-acting corticosteroids with the highest glucocorticoid efficacy and a biological half-life of 36 to 54 h. Cortisone and cortisol, on the other hand, are short-acting with a half-life of less than 12 h and are less commonly used. Prednisone, prednisolone, methylprednisolone, and triamcinolone fall into the intermediate-acting category with a half-life of 18 to 36 h. Some adverse effects of corticosteroids follow a linear dose–response pattern, where the incidence increases with higher doses, while others exhibit a threshold dose–response pattern, with a higher frequency of events beyond a specific threshold value [95,97].

The metabolism of midazolam, a benzodiazepine drug, may potentially be enhanced in patients receiving long-term treatment with corticosteroids. The simultaneous use of these two medications can lead to adverse effects, such as central nervous system depression, resulting in drowsiness, decreased motor coordination, difficulty in speech and reasoning, and impaired motor skills. These effects can be particularly risky for elderly or more vulnerable patients who are more prone to complications and increased risks.

Moreover, the interaction between benzodiazepines and corticosteroids can affect the absorption, distribution, and elimination of these drugs as well as other medications the patient may be taking. This can lead to unexpected blood levels, either higher or lower than anticipated, thereby increasing the risk of side effects. Benzodiazepines, phenobarbital, and meprobamate selectively inhibit the rise of plasma corticosteroid levels, whereas other psychotropic drugs such as tricyclic antidepressants, monoamine oxidase inhibitors, neuroleptics, and amphetamines do not have this effect. This impact of benzodiazepines on corticosteroid levels is centrally mediated since diazepam does not block the increase in plasma corticosteroid levels caused by adrenocorticotropic hormone [97,98].

## 4. Discussion

Every drug has the potential of interacting with another, and that should be one of the main factors to have in count when deciding and planning surgeries, since these drugs can change the whole dynamics of the perioperative period. It is well established that the perioperative period is incredibly complex and challenging for patients, healthcare teams, anesthetists, and surgeons. Pharmacological treatment plays a vital role in ensuring the safety and effectiveness of surgical procedures, highlighting the significance of DDIs. Recognizing the impact of DDIs on treatment efficacy and safety is crucial.

These interactions can affect the efficacy and safety of drugs administered during surgery, potentially resulting in unwanted effects, decreased treatment efficacy, or even risks to the patient. An in-depth understanding of DDIs is essential to avoid potential problems and ensure proper use of medications during the perioperative period. A notable example of drug interactions in the context of anesthesia is propofol, one of the most commonly used anesthetics. Propofol is often administered in combination with other anesthetic drugs such as remifentanil, isoflurane, fentanyl, and sevoflurane; however, these combinations may result in significant drug interactions. Propofol may potentiate the respiratory and cardiovascular depressant effects of remifentanil and may interact with inhaled anesthetic agents such as isoflurane and sevoflurane, which may lead to additive or synergistic effects on central nervous system depression, increasing the risk of excessive sedation and respiratory depression. Another important drug interaction involves fentanyl, an opioid analgesic used in combination with propofol during anesthesia, and coadministration of propofol and fentanyl may result in a potentiation of sedative and analgesic effects.

DDIs can affect various several mechanisms in the body, altering absorption, distribution, metabolism, and excretion of the drugs. Therefore, it is important to be aware of possible drug interactions and take steps to minimize their risk. First and foremost, reviewing the patient’s medication list and identifying drugs that may interact based on literature to consider the dosage and duration of medications that will be administered during the perioperative period can be of extreme importance. Thus, it may be necessary to adjust the dosage or change the medication to minimize the risks of interactions. Second, it is important to monitor the patient before, during, and after the surgical procedure to help detect any signs of drug interactions and complications, which include monitoring heart rate, blood pressure, and blood oxygen levels, as well as monitoring plasma levels of medications. Several significant considerations come into play when assessing DDIs in the perioperative period: (1) recognizing the pharmacodynamic effects of commonly used chronic medications during elective surgeries is crucial. These include cardiovascular drugs, lipid-lowering drugs, gastrointestinal medications, pulmonary medications, antibiotics, opioids and non-opioid analgesics, gabapentanoids, erectile dysfunction drugs, and psychotropic drugs. (2) Conducting a risk assessment of the patient population helps identify individuals at higher risk for DDIs based on factors such as age, comorbidities, genetic variations, and polypharmacy. (3) Recognizing the half-life of routinely used medications and adjusting the dosage according to the perioperative schedule is crucial to maintaining therapeutic levels during surgery. (4) Obtaining a comprehensive medical history and involving all clinicians in patient management (e.g., surgeon, anesthesiologist, and medical consultant) ensures a thorough understanding of the patient’s background, enabling the identification of potential DDIs.

One of the approaches to take in consideration when discovering and preventing pharmacological interactions in the perioperative period involves reviewing the existing medical literature to identify potential interactions proactively. This includes examining scientific articles, systematic reviews, and meta-analyses that investigate the effects of medications used during this period. However, by applying precision medicine principles, clinicians can better understand the pharmacodynamic effects of commonly used chronic medications during elective surgeries. Conducting pharmacokinetic and pharmacodynamic studies in patients taking multiple medications during surgery can help identify interactions that may impact drug efficacy and safety. Real-time drug interaction alert systems in hospital environments aid in recognizing and avoiding dangerous drug combinations. Collaborative adverse event monitoring, including reviewing reports of adverse events and medication errors, contributes to patient safety.

At last, conducting pharmacokinetic and pharmacodynamic simulations in patients undergoing surgery who are taking multiple medications aids in recognizing potential pharmacological interactions that may impact the safety or effectiveness of drugs administered during the perioperative period. To further enhance this process, real-time drug interaction alert systems can be employed during the prescription and administration of medications within the hospital environment. These systems play a vital role in identifying drug interactions and generating alerts for doctors and nurses, allowing them to avoid potentially dangerous drug combinations. Moreover, collaborative adverse event monitoring, encompassing the review of adverse event reports and medication error notifications, serves as an additional method for identifying DDIs and promoting patient safety.

Another valuable approach to prevent DDIs is the utilization of pharmacokinetic simulations conducted in laboratory settings. These simulations help predict potential pharmacological interactions beforehand between drugs a patient is taking, assisting in determining appropriate drug dosages during the perioperative period and thereby minimizing the occurrence of undesirable pharmacological interactions.

## 5. Conclusions

Drug–drug interactions (DDIs) play a crucial role in shaping the dynamics of surgery, underscoring the significance of conducting patient-specific investigations to identify appropriate medications and anticipate potential interactions. By employing the strategies discussed in this review, healthcare professionals can enhance their ability to recognize and manage DDIs during the perioperative period, thereby minimizing risks and optimizing patient outcomes. The use of pharmacokinetic and pharmacodynamic simulations, real-time drug interaction alert systems, collaborative adverse event monitoring, and pharmacokinetic simulations in the laboratory all contribute to the prevention of DDIs. By integrating these methods into clinical practice and considering their findings, healthcare providers can improve patient safety and reduce the occurrence of DDIs in future surgical interventions. This tailored approach to identifying and addressing DDIs ensures a more personalized and effective treatment regimen for each patient, ultimately leading to better surgical outcomes.

## Figures and Tables

**Table 1:** A scheme that includes all the drugs used in the three phases of the perioperative period. These drugs, especially intraoperative anesthetics, are FDA approved. The arrows represent the type of interaction that the drug presents. If it is a vertical interaction with other drugs, then the arrow is vertical, and when drugs present horizontal interactions with other drugs, then the arrow is horizontal. Drugs that interact with other drugs are written in bold.

Preoperative Phase	Intraoperative Phase	Postoperative Phase
Corticosteroids	Alfentanil	**Benzodiazepine ↔↕**
**Benzodiazepine ↔↕**	Articaine	**Celecoxib ↕**
Dipyrone	Benzocaine	**Corticosteroids ↕**
Gabapentin	Butorphanol	**Ibuprofen ↕**
**Methadone ↕**	Cisatracurium	**Morphine ↕**
Midazolam	Desflurane	**Methadone ↔**
**Paracetamol ↕**	Epinephrine	Paracetamol
**Tramadol ↕**	Etomidate	
	**Fentanyl ↕**	
	Glycopyrrolate	
	Hyoscyamine	
	**Isoflurane ↕**	
	**Ketamine ↔↕**	
	Lidocaine	
	Methohexital	
	Nalbuphine	
	Prilocaine	
	**Propofol ↕**	
	**Remifentanil ↕**	
	Remimazolam	
	**Rocuronium ↕**	
	**Sevoflurane ↕**	
	Succinylcholine	
	Sufentanil	
	**Vecuronium ↕**	

Drugs that interact with other drugs are written in bold. The horizontal arrow (red) means the interaction between drugs from different phase (columns). The vertical arrow (blue) means interactions in the same column (same phase).

## Data Availability

Not applicable.
